# A Late Devonian tree lycopsid with large strobili and isotomous roots

**DOI:** 10.1038/s42003-022-03934-4

**Published:** 2022-09-15

**Authors:** Le Liu, De-Ming Wang, Yi Zhou, Min Qin, David K. Ferguson, Mei-Cen Meng

**Affiliations:** 1 School of Geoscience and Surveying Engineering, China University of Mining and Technology (Beijing), Beijing, 100083 China; 2 Key Laboratory of Orogenic Belts and Crustal Evolution, School of Earth and Space Sciences, Peking University, Beijing, 100871 China; 3 Institute of Geology and Paleontology, Linyi University, Linyi, 276000 China; 4 Department of Palaeontology, University of Vienna, Vienna, 1090 Austria; 5 Science Press, China Science Publishing & Media Ltd., Beijing, 100717 China

**Keywords:** Palaeontology, Plant evolution

## Abstract

Tree lycopsids prospered in the Late Devonian and constituted a major part of the Late Paleozoic forest ecosystem that deeply impacted the Earth’s climate. However, the fertile organs of these early tree lycopsids display low morphological disparity, which has hampered further knowledge about their ecological habit. Here, we report *Omprelostrobus gigas* gen. et sp. nov. from the Upper Devonian (Famennian) Wutong Formation at Changxing, Zhejiang, China. The collection includes aerial axes, strobili and associated roots. The strobili are the largest among coeval lycopsids to our knowledge, and are divided into proximal and distal portions by dimorphic sporophylls with differentiated laminae and probable strong photosynthetic capacity. The associated but not attached roots displaying multiple isotomous branches lack rootlets and typical rootlet scars. The varied strobili sizes of early tree lycopsids were relatively independent of their body plan, but the large strobili could suggest increased reproductive investment to overcome the disadvantages of the disturbed flooded habitat.

## Abstract (web summary)

A new species of Late Devonian fossil lycopsid with large strobili and differentiated sporophylls provides insight into reproductive investment of early tree lycopsids.

## Introduction

The development of Late Paleozoic forests considerably changed the Earth’s climate by accelerating silicate weathering and increasing organic carbon burial, which resulted in a great consumption of atmospheric CO_2_ and a major icehouse period^1–4^. The earliest forests consisted of trees belonging to the cladoxylopsids (fernlike plants) and the progymnosperms in the Middle Devonian^5,6^, as well as the lycopsids and probably progymnosperms in the Late Devonian^7–10^. Lycopsids are regarded as one of the earliest diverging groups of vascular plants that could be traced back to the late Silurian^11^. During the Middle and Late Devonian, arborescent lycopsids evolved key characteristics for the tree habit including centralized rooting systems^10,12^ and thick stems with secondary growth^13,14^. These tree lycopsids contributed to the formation of the earliest forests, which played important roles in CO_2_ decline, coastal consolidation, and the weathering process/soil formation^9,10^. Tree lycopsids reached huge sizes and dominated the Carboniferous swamp forests^15^. These giants were supported by enormous rootstocks^16^ and had trunks 30–40 m long and 2 m in diameter^17,18^, bearing strobili up to 1 m long^19^. Although the fossil record suggests an increased body size in many Devonian tree lycopsids^20,21^, significant changes in strobili size and structure are lacking in these taxa^22–25^. Such biological conservatism suggests uniform reproductive habit/strategy of Late Devonian tree lycopsids, which deserves further investigation.

In this article, a new lycopsid *Omprelostrobus gigas* gen. et sp. nov., is described from the Upper Devonian in Zhejiang Province, South China. This plant displays the largest strobili among coeval taxa, and wide axes associated with isotomous roots, providing knowledge on the characteristic evolution and ecological habit of Devonian tree lycopsids.

## Results

### Locality and stratigraphy

The fossil plant was excavated from the Upper Devonian at the Fanwan section, Hongqiao Town, Changxing County, Zhejiang Province, China. Detailed locality and exposed strata have been illustrated by Wang et al.^23^. At the Fanwan section, the Wutong Formation is composed of two members in ascending order, i.e., the Guanshan Member with massive quartz sandstone and a few argillaceous siltstone intercalations, as well as the overlying Leigutai Member with interbedded quartz sandstone and silty mudstone. Fossil plants occur in the 13th bed of the Fanwan section in the upper part of the Leigutai Member, from which the specimens of *Cosmosperma polyloba* and *Changxingia* sp.^26–28^. were collected. However, only *C*. *polyloba* branches and several isotomous roots are found closely associated with the current fossil lycopsid. The age of the upper part of the Leigutai Member is regarded as the latest Famennian based on the LC (*Knoxisporites literatus*-*Reticulatisporites cancellatus*) spore assemblages^29^.

### Systematic paleobotany

**Class** Lycopsida Pichi-Sermolli 1958

**Order** Isoёtales *sensu lato* DiMichele & Bateman, 1996

**Suborder & Family** Incertae sedis

**Genus** *Omprelostrobus* gen. nov. Liu, Wang, Zhou, Qin, Ferguson, and Meng.

#### Etymology

The generic name comes from greek “*omprelo*-” (umbrella) and “-*strobus*” (cone), referring to the pendulous strobili with long sporophylls radiating proximally.

#### Generic diagnosis

Tree lycopsid with arial axes and large strobili. Long-fusiform leaf cushions helically arranged along axes. Strobili single, terminating a fertile axis. Each strobilus divided into proximal and distal portions based on sporophyll morphology: the proximal portion with sporophylls bearing long, radiating laminae; the distal portion characterized by sporophylls with short, adpressed laminae. A single sessile long ellipsoidal sporangium borne on the adaxial surface of the sporophyll pedicel.

**Type species** *Omprelostrobus gigas* gen. et sp. nov. Liu, Wang, Zhou, Qin, Ferguson, and Meng.

#### Etymology

The specific epithet is from greek “*gigas*” (gigantic), referring to the large size of the strobili.

#### Holotype designated here

PKUB19306

#### Paratypes

PKUB19301-PKUB19305, PKUB19307-PKUB19309, PKUB19310A, B, PKUB19311.

#### Specific diagnosis

Tree lycopsid with aerial axes and large strobili. Preserved axes up to 8.0 cm wide. Helically arranged leaf cushions long fusiform, with length/width ratio ranging from 9.2–11.6:1. Each leaf cushion displaying an oblanceolate leaf scar in the middle. A single strobilus, 5.4–28.2 cm long and 9.3–51.2 mm wide (the length/maximum width ratio ranging from 5.5–6.5:1), singly terminating a fertile axis up to 12.4 cm long and 3.9–6.1 mm wide. The proximal portion of the strobili, 6.4–10.8 cm long and 16.6–51.2 mm wide (the length/width ratio ranging from 2.1–3.1:1), bearing sporophylls with linear, radiating laminae 3.7–5.7 cm long. The distal portion of strobili bearing adpressed sporophylls with lanceolate laminae 0.7–2.4 cm long and bent adaxially. Sporophyll lamina possesses a strong midvein. The strobilar axes 2.5–8.8 mm wide. A single sessile sporangium, long ellipsoidal, 5.2–10.3 mm long and 1.5–1.8 mm high (the length/height ratio ranging from 3.5–6.4:1), presented on the adaxial surface of the sporophyll pedicel.

#### Repository

Department of Geology, Peking University, Beijing, China.

#### Type locality

Fanwan Village, Hongqiao Town, Changxing County, Zhejiang Province, China.

#### Horizon and age

Wutong Formation, Late Devonian (Famennian).

### Description

**Fig. 1:**
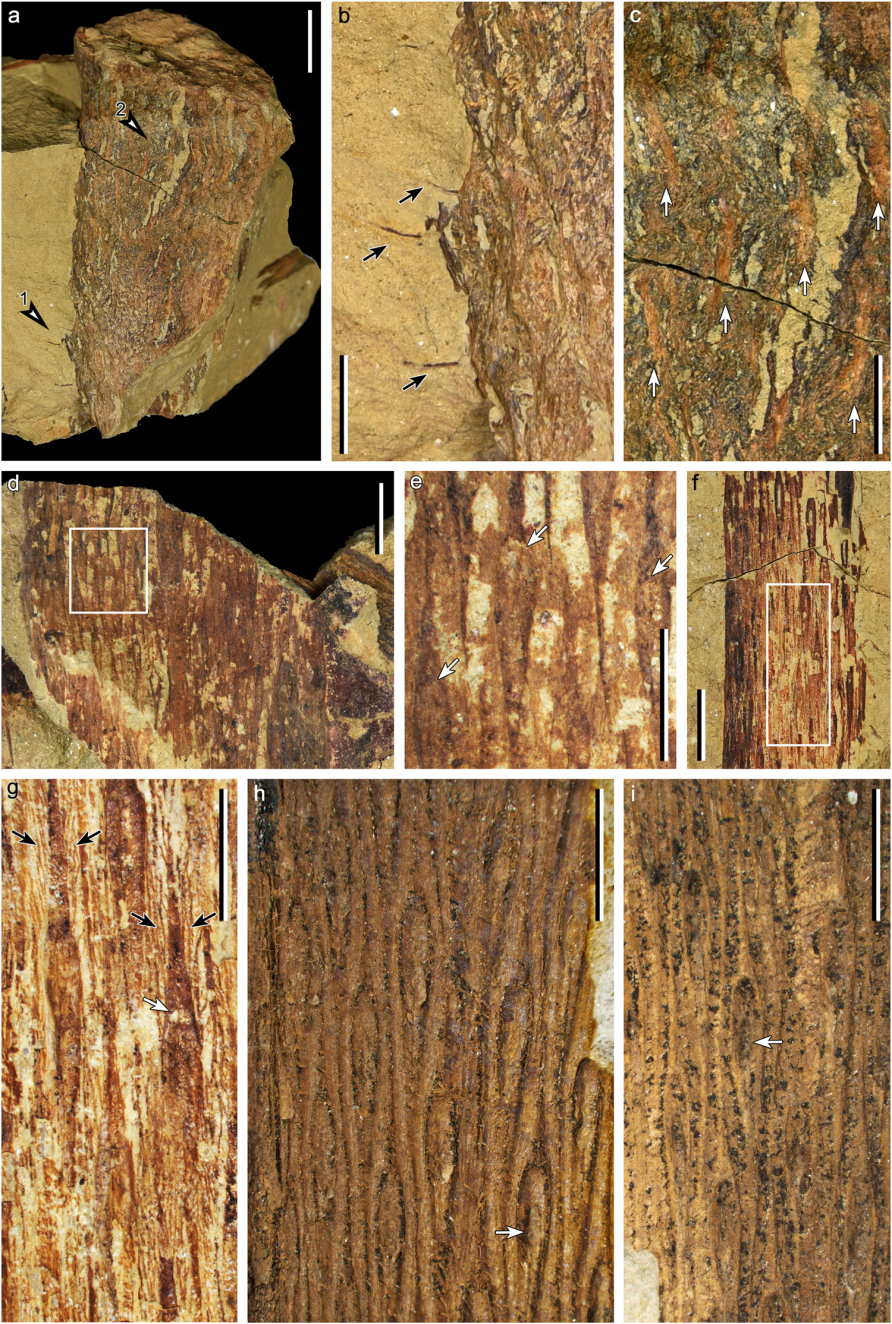
Aerial axes bearing leaf cushions of *Omprelostrobus gigas* gen. et sp. nov. Scale bars: a, 2 cm; b–d, f, 1 cm; e, g–i, 5 mm. **a** Axis cast crossing the bedding plane. Arrows 1 and 2 indicating portion enlarged in **b** and **c**, respectively. PKUB19301. **b** Enlargement of **a** (arrow 1), displaying leaf remains (arrows). **c** Enlargement of **a** (arrow 2), displaying twisted vestiges of leaf cushions/bases (arrows). **d** A thick axis displaying leaf cushions. Rectangle indicating portion enlarged in **e**. PKUB19302. **e** Enlargement of **d**, displaying fusiform leaf cushions with interspaces. Arrows indicating the arched sutures suggesting the upper margins of leaf scars. **f** An axis displaying leaf cushions. Rectangle indicating portion enlarged in **g**. PKUB19303. **g** Enlargement of **f**, displaying fusiform leaf cushions and striate ornamentations (paired black arrows) in interspaces. White arrow indicating the arched suture suggesting the upper margin of a leaf scar. **h** Enlargement of Fig. 4b (arrow), displaying helically arranged fusiform leaf cushions with oblanceolate leaf scars and oval vascular bundle scar (arrow). PKUB19304. **i** Axis displaying helically arranged fusiform leaf cushions with oblanceolate leaf scars. Arrow indicating probable vascular bundle scar. PKUB19305.

**Fig. 2:**
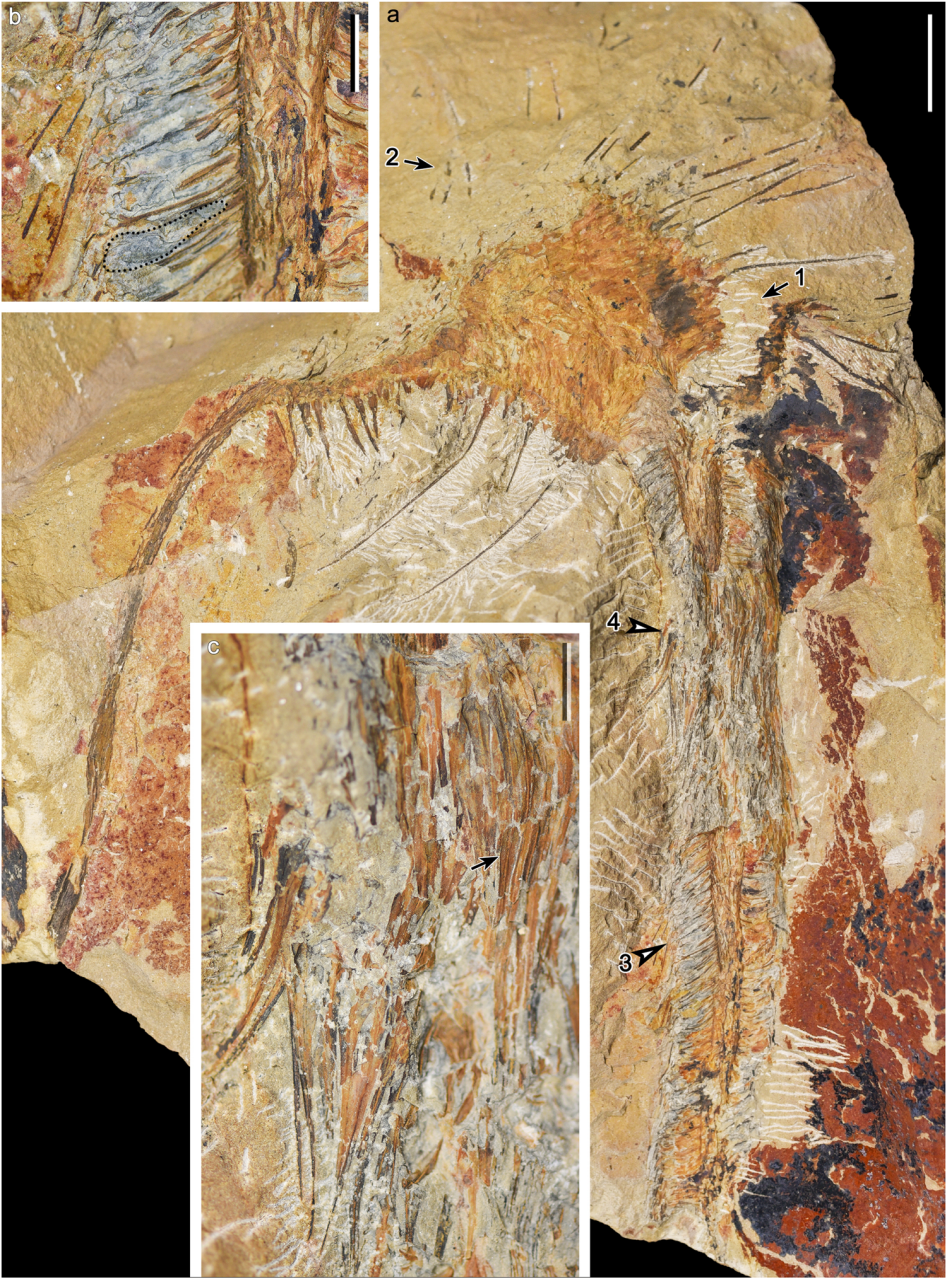
The type specimen of *Omprelostrobus gigas* gen. et sp. nov. Scale bars: a, 2 cm. b, c, 5 mm. **a** Strobilus terminating the fertile axis. Arrow 1 indicating fractures near the boundary between the proximal and distal portions, and arrow 2 indicating sporophylls in rock matrix. Arrows 3 and 4 indicating portions enlarged in **b**, **c**, respectively. PKUB19306. **b** Enlargement of **a** (arrow 3), displaying the strobilar axis and sporophylls on one side. Dotted line indicating the contour of a sporangium. **c** Enlargement of **a** (arrow 4), displaying sporophyll laminae in face view. Arrow indicating laminae with strong midvein.

**Fig. 3:**
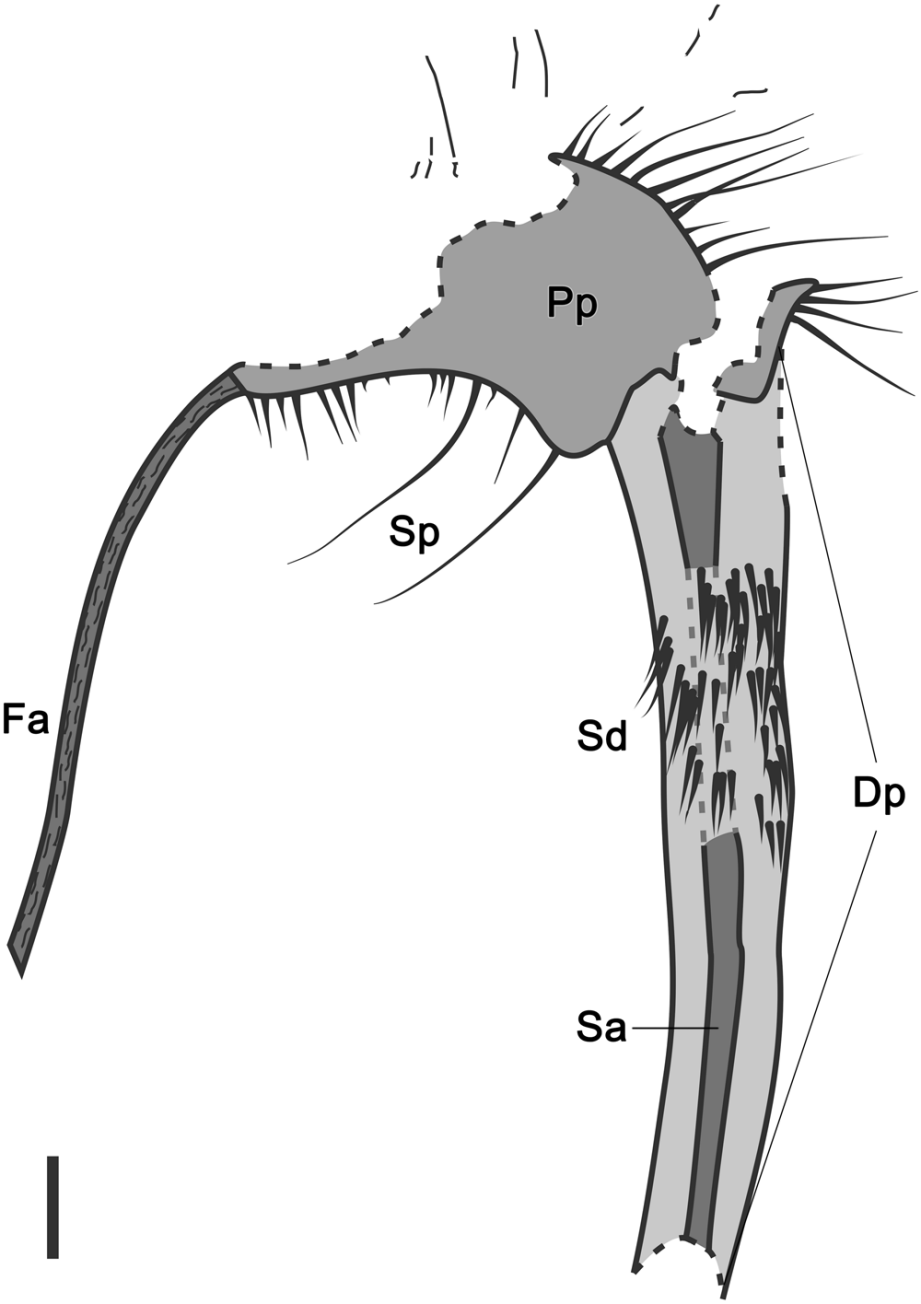
Interpretative line drawing of the strobilus in the type specimen. Fa fertile axis, Pp proximal portion of the strobilus, Dp distal portion of the strobilus, Sp Sporophylls on the proximal portion, with long, radiating laminae, Sd Sporophylls on the distal portion, with shorter adpressed laminae pointing towards the tip of strobilus. Sa strobilar axis. Gray dashed lines indicating the covered part of strobilar axis. Black dashed lines indicating the fractures. Scale bar: 2 cm.

**Fig. 4:**
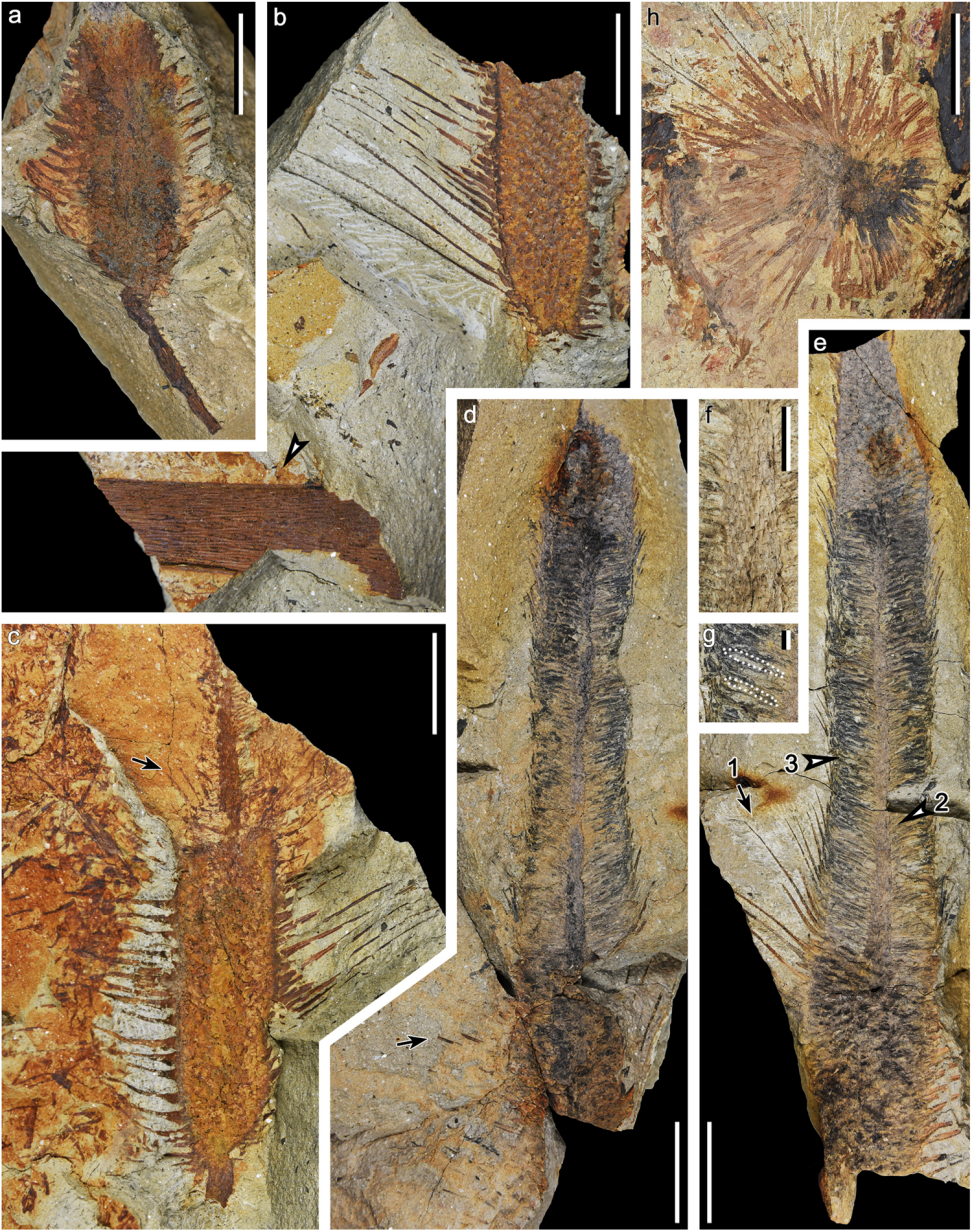
Strobili of *Omprelostrobus gigas* gen. et sp. nov. Scale bars: a–e, h, 2 cm; f, 5 mm, g, 2 mm. **a** A strobilus terminating fertile axis. PKUB19307. **b** Strobilus displaying long linear sporophyll laminae along one side. Arrow indicating associated axis enlarged in Fig. 1h. **c** A strobilus terminating fertile axis, displaying sporophyll laminae along both sides. Arrow indicating the sporophyll laminae shorten and bend towards the tip of the strobilus at the boundary between the proximal and distal portions. PKUB19308. **d**, **e** Part and counterpart of a strobilus. Arrow in **d** indicating sporophylls in rock matrix. Arrow 1 in **e** indicating the sporophyll laminae shorten and bend towards the tip of the strobilus at the boundary between the proximal and distal portions. Arrows 2 and 3 in **e** indicating portions enlarged in **f**, **g**, respectively. PKUB19309A, B. **f** Enlargement of **e** (arrow 2). Strobilar axis with scars of sporophyll pedicels. **g** Enlargement of **e** (arrow 3). Dotted lines indicating contours of sporangia. **h** Probable strobilus with radiating sporophylls cutting across the bedding plane. PKUB19310.

**Fig. 5:**
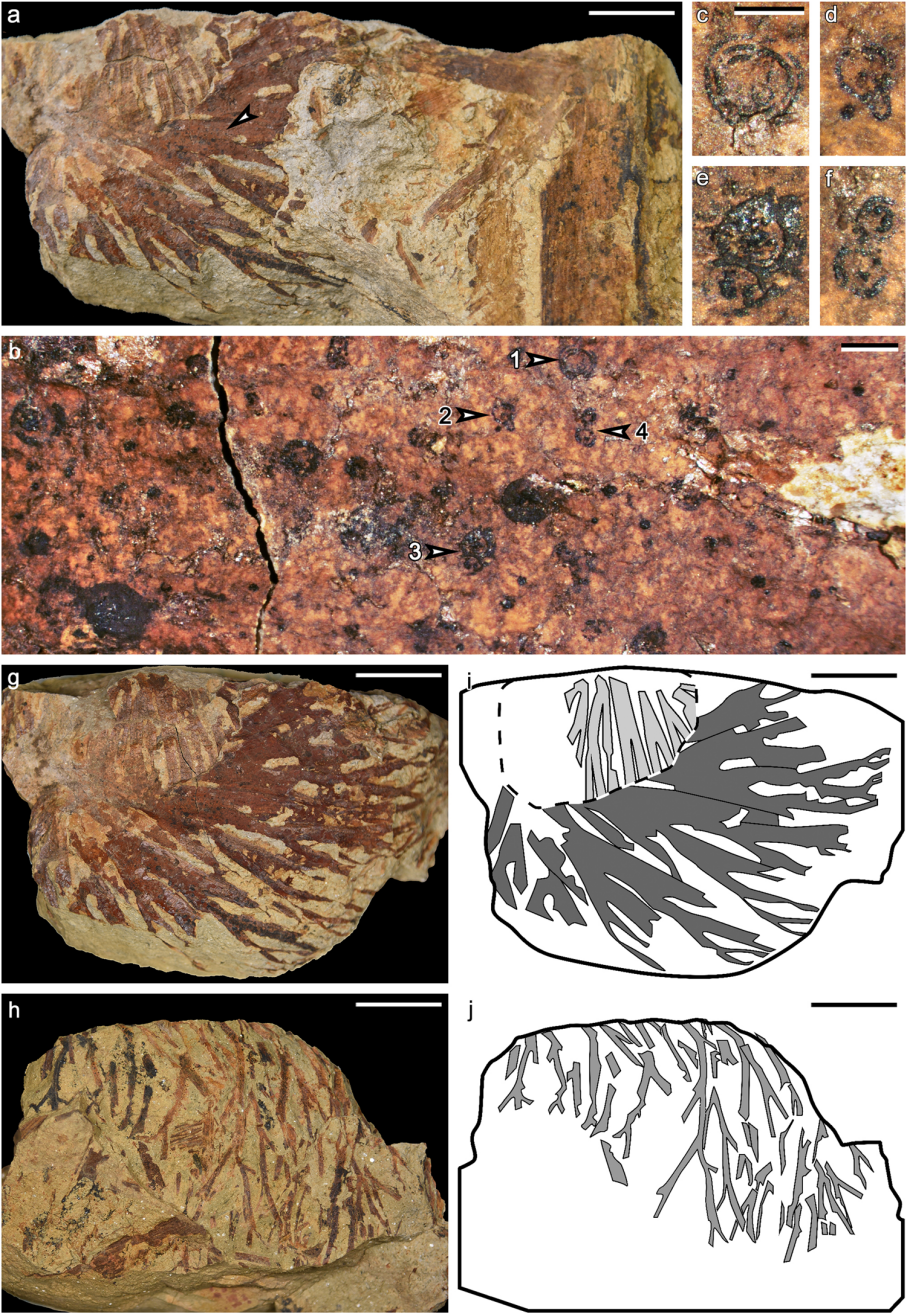
Associated roots of *Omprelostrobus gigas* gen. et sp. nov. Scale bars: a, g–j: 2 cm; b, 1 mm; c–f, 0.5 mm. **a** Dichotomized roots radially arranged in two directions, associated with a lycopsid axis. Arrow indicating portion enlarged in **b**. PKUB19311. **b** Enlargement of **a** (arrow), showing the arrangement of circular structures. Arrow 1–4 indicating circular structures enlarged in **c**–**f**, respectively. **c**–**f** Enlargement of **b** (arrow 1–4), respectively, showing details of the circular structures. **g** Roots shown in **a**, after dégagement, showing multiple dichotomies. **h** The other side of specimen shown in **g**, showing more slenderer roots with multiple dichotomies. **i**, **j** Interpretative line drawings of the roots shown in **g**, **h**, respectively. Grayscale indicates roots in different orientations.

About 30 specimens containing plant organs of *Omprelostrobus gigas* have been studied. These specimens display vegetative aerial axes of varied diameters (Fig. 1), fertile axes with terminal strobili (Figs. 2–4), and isotomous roots closely associated with the shoot (Fig. 5).

#### Vegetative axes

An axis cast (Fig. 1a) transversing the bedding plane shows a preserved length and diameter of 11.7 cm and 8.0 cm, respectively. It has only two or three possible leaf remains (Fig. 1a, arrow 1, 1b, arrows), but several twisted vestiges of leaf cushions/bases along its surface (Fig. 1a, arrow 2, 1c, arrows). Other vegetative axes are mostly preserved as adpressions, displaying leaf cushions but having no leaves attached (Fig. 1d–i). A 4.3 cm wide axis exhibits helically arranged and long-fusiform leaf cushions (Fig. 1d, e) that are ca. 16.3 mm long and 1.4 mm wide (length/width ratio = 11.6:1, *n* = 8). The interspaces between leaf cushions are ca. 1.1 mm wide. Leaf cushions with similar dimensions and helical arrangement occur on another axis of 4 cm wide (Fig. 1f), while the interspaces between the leaf cushions are filled with striate ornamentations (Fig. 1g, paired black arrows). The angle between parastichies of leaf cushions and horizontal lines is ca. 70°. An arched suture occurs within these leaf cushions (Fig. 1e, arrows; g, white arrow) suggesting the upper margins of leaf scars, while their lateral margins merge with those of the leaf cushions. On slenderer axes of ca. 10 mm wide, the leaf cushions measure ca. 12.0 mm long and ca. 1.3 mm wide (length/width ratio = 9.2:1, *n* = 6), with each cushion having an oblanceolate leaf scar in the middle (Fig. 1h, i, Supplementary Fig. 1). The leaf scars are ca. 4.5 mm long. An oval protrusion, 1.8 mm long and 0.7 mm wide, is located in the mid-lower part of the leaf scar (Fig. 1h, arrow), and is interpreted as the vascular bundle scar (Supplementary Fig. 1, Vbs); correspondingly, a similar structure is shown as a long elliptical concavity in the leaf cushion on a cast of an axis (Fig. 1i, arrow). However, comparable structures on many other leaf scars, as well as the existence of ligule pits, cannot be distinguished due to poor preservation. All these axes display neither dichotomies nor lateral branches.

#### Strobili

A large strobilus terminates a slender fertile axis 12.4 cm long and 4.4 mm wide (Fig. 2a). The axis width does not change significantly along its length. The fertile axis shows neither branching, nor attached leaves, nor any clear leaf bases along the surface. The strobilus, which is the largest and the best-preserved in the collection, measures ca. 28.2 cm long with its distal end missing. It is 20.3–51.2 mm wide excluding the sporophylls, and can be clearly divided into proximal and distal portions based on the dimorphic sporophylls (Fig. 3). On the right side near the boundary between the proximal and distal portions the strobilus is fractured (Fig. 2a, arrow 1), as shown by a repetition on both sides of the fracture.

The proximal portion of the strobilus is curved and up to 10.8 cm long. Its lower part is broken but could be inferred by dispersed sporophylls in the rock matrix (Fig. 2a, arrow 2). Sporophylls on the proximal portion show linear and radiating laminae up to 5.7 cm long, and forming an angle of 70–90° with the strobilus. The sporophylls are ca. 1.8 mm at their bases and ca. 0.5 mm wide near the tips (*n* = 8). The strobilar axis and the details of sporangia cannot be recognized on the proximal portion due to the dense sporophylls.

The distal portion of the strobilus is almost straight and 17.4 cm long. The width of the distal portion tapers acropetally, from 31.9 mm at base to 20.3 mm at the distalmost part. Sporophylls borne distally are densely arranged along the strobilar axis 4.7–8.8 mm in width. Each sporophyll comprises a slightly oblique or nearly horizontal pedicel (Fig. 2a, arrow 3, 2b), and a lamina bent adaxially towards the tip of strobilus, unlike those sporophylls born on the proximal portion of the strobilus. The pedicels are 8.2–10.3 mm long (*n* = 6), and each bears a long ellipsoidal sporangium on the adaxial surface; the sporangium is 7.7–8.0 mm long and 1.5–1.8 mm high (Fig. 2b, dotted line, n = 7). The sporophyll laminae are lanceolate in front view with entire margins, 1.0–2.4 cm long and 1.9 mm at the widest part (Fig. 2a, arrow 4, 2c, *n* = 8), with each lamina possessing a strong midvein (Fig. 2c, arrow).

Another eight fossils illustrate strobili of the same kind as the type specimen. However, they are usually broken at proximal or distal ends, with the preserved lengths ranging from 5.4–17.6 cm. Three strobili (Fig. 4a–c), and one strobilus preserved in part and counterpart (Fig. 4d, e), largely display the “proximal portions” and “distal portions” of the strobili, respectively. Two of these strobili are attached to the fertile axes (Fig. 4a, c), bearing neither leaves nor clear leaf bases. The fertile axes have a maximum preserved length of 3.4 cm and are 3.9–6.1 mm wide, and the widths do not change significantly along their lengths. The proximal portions of these strobili are maximum 16.6–26.8 mm in width excluding the sporophylls, and one strobilus displays the whole length of its proximal portion of 6.4 cm (Fig. 4c). Sporangia and strobilar axes cannot be recognized in these proximal portions. The long linear sporophyll laminae on the proximal portions of the strobili (Fig. 4b, c, arrow in 4d) radiate and display similar dimensions to those of the type specimen (Fig. 2a). However, at the boundary between the proximal and distal portions, the sporophyll laminae shorten and gradually bend towards the tip of the strobili (Fig. 4c, arrow, 4e, arrow 1). The distal portions of the strobili in Fig. 4c–e reach 12.2 cm long and are 9.3–25.0 mm wide, with the width tapering acropetally. The strobilar axes are 2.5–4.2 mm wide (Fig. 4c–e) and the sporophylls are densely arranged in helices (Fig. 4e, arrow 2, 4f). These sporophylls possess adpressed laminae 0.7–1.2 cm long (*n* = 12). The sporangia attached to the sporophyll pedicels are elongate ellipsoidal (Fig. 4e, arrow 3, 4g, dotted lines), 5.2–9.6 mm long, and ca. 1.5 mm high (*n* = 16).

One strobilus probably in transverse fracture illustrates radially extended laminae along the bedding plane (Fig. 4h). These laminae are linear, 3.7–5.4 cm long and ca. 1.3 mm wide at base (*n* = 10), and fall within the dimension range of sporophylls on the proximal portion of a strobilus.

Spores in all these strobili are badly preserved and cannot be identified. The anatomy is unknown.

#### Associated roots

Repeatedly isotomous organs are preserved oblique to the bedding plane of the rock and are thus considered as roots (Fig. 5a). They are closely associated with lycopsid axes but not attached to them. Tiny circular structures occasionally occurred at basal parts of some root (Fig. 5b). They are 0.3–1.0 mm in diameter, and displayed varied appearance including solid or multilayered concentric circles, with papilla, or in pairs (Fig. 5b–f). On two sides of the specimen, roots in three orientations were revealed after dégagement (Fig. 5g–j) and may represent three different groups adjacent to each other. One group of roots in a radial pattern (Fig. 5g; marked with dark gray in 5i) indicates that they may have been closed to and diverged from the base of the plant. Preserved roots are up to 5.6 cm long and the thickest root branches are ca. 6.5 mm wide. The width decreases acropetally, with the thinnest distal branches being 1.0 mm wide (Fig. 5g–j). One root could dichotomize as many as five times, with the branch intervals varying from 0.3 to 1.8 cm. No rootlet or rootlet scar have been observed along these roots.

### Comparisons

#### Vegetative characteristics

Considering the widest axes of 8.0 cm, *Omprelostrobus gigas* is most likely to have been an arborescent plant. Its arial axes display leaf cushions. Among the other Devonian arborescent lycospsids, *Jurinodendron kiltorkense* (previously known as *Cyclostigma kiltorkense*) has rounded leaf scars with intrafoliar parichnos scars, but lacks a typical leaf cushion^30^, unlike *O*. *gigas* and most other coeval taxa. Probably due to the absence of an abscission layer at the base of the leaves, both thick stems and slender axes of *Sublepidodendron songziense* and *S*. *grabaui* only exhibit a fissure-like “false leaf scar” at the leaf bases^14,24^, and hence differ from *O*. *gigas*. The *O*. *gigas* leaf cushions are long fusiform, differing from those rhombic to quadrate ones in *Leptophloeum rhombicum*^31^, oblanceolate cushions in *Changxingia longifolia*^23^ and oval ones in *Changxingia* sp.^28^. *Minostrobus chaohuensis* displays striate ornamentations between leaf cushions resembling that of *O*. *gigas*, while its leaf cushions are shorter and the vascular bundle scar is closer to the top of the leaf scar^32^. *Guangdedendron micrum* bears fusiform leaf cushions^33^, differing from those of *O*. *gigas* in their wider shape and the presence of an obvious rounded leaf scar in the middle.

#### Fertile organs

We have listed some typical Devonian lycopsid taxa in Table 1 and compared the dimensions of their fertile structures. Most of these strobili are not more than 160 mm in length and 10 mm in width. *Longostachys latisporophyllus*, *Guangdedendron micrum,* and *Omprelostrobus gigas* have strobili over 200 mm long, and *O*. *gigas* exhibits the longest of 282 mm. On the other hand, the taxa possessing an unbranched trunk, *Clevelandodendron ohioensis* and probably *Cymastrobus irvingii*, display the widest but short strobili of ca. 60 mm in width. The preserved maximum width of *O*. *gigas* strobili reaches 51.2 cm and is the third widest among these taxa, clearly ahead of the fourth *G*. *micrum* that is ca. 30 mm. Thus, *O*. *gigas* had the largest strobili among these lycopsids. In addition, *O*. *gigas* also possessed sporophyll laminae longer than those of most other lycopsid plants, while its dimorphic sporophylls in a single strobilus are unique.

**Table 1:** The dimensions of some representative Devonian lycopsids and their fertile structures.

Taxon	Age	Maximum axes width (cm)	Possible height (m), calculation based on Mosbrugger^53^	Length of strobili (mm)	Width of strobili (mm)	Length of sporophyll laminae (mm)
*Yuguangia ordinata*^22^	Givetian	1.0	1.1	160	8.6	8–10
*Longostachys latisporophyllus*^13^	Givetian	3.5	2.5	30–225	7–10	15–30
*Kossoviella timanica*^54^	Frasnian	2.0	1.7	50–160	2–8	8–18
*Lilingostrobus chaloneri*^25^	Famennian	0.5	0.7	56	7	50
*Wuxia bistrobilata*^39^	Famennian	1.4	1.4	105	13	55
				30	22	95
*Clevelandodendron ohioensis*^38^	Famennian	2.0	1.7 (1.25 in fully preserved specimen)	90	60	18
*Cymastrobus irvingii*^55^	Famennian	/	/	75	58	/
*Changxingia longifolia*^23^	Famennian	2.0	1.7	20–50	6.0–9.6	12–18
*Changxingia* sp.^28^	Famennian	1.2	1.2	10.0–34.8	5.0–8.8	15.2
*Minostrobus chaohuensis*^32,56^	Famennian	5.5	3.4	80	6.0	5.0
				125	4.5–6.0	6.0–7.0
*Sublepidodendron songziensis*^14,57^	Famennian	7.0	4.0	80–120	8.0–12	1.0–6.0
				100–150	6.0–10.0	
*S*. *grabaui*^24,58^	Famennian	10.0	5.1	90	8.0–10	/
				160	8.0	10
*Guangdedendron micrum*^10,33^	Famennian	18.7	7.7	50–234	9–30	5.3*–18
*Omprelostrobus gigas*	Famennian	8.0	4.4	54–282	17.6–51.2	10–54

*Data measured from illustrated figures or plates.

## Discussion

### Dimensions and growth pattern of Devonian lycopsid fertile structures

**Fig. 6:**
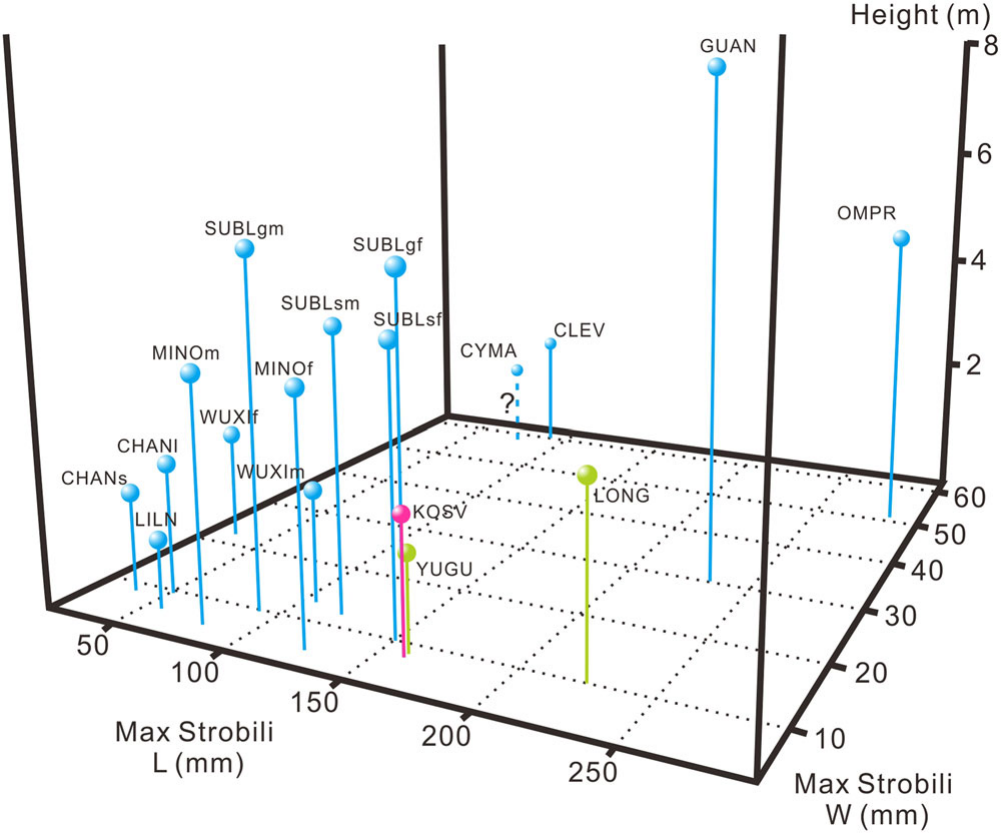
3-D plot of the maximum strobilus lengths (mm), maximum strobilus widths (mm), and the calculated maximum plant height (m, according to Mosbrugger^53^) of some representative Devonian lycopsids. Olivaceous, pink, and blue pins indicate the Givetian, Frasnian, and Famennian ages, respectively. Pin in dashed line with a question mark “?” indicating unknown plant height. Data in this diagram according to Table 1. Abbreviations (from left to right): CHANs: *Changxingia* sp.; LILN: *Lilingostrobus chaloneri*; CHANl: *C*. *longifolia*; MINOm: *Minostrobus chaohuensis* (microsporangiate strobili); WUXIf: *Wuxia bistrobilata* (megasporangiate strobili); SUBLgm: *Sublepidodendron grabaui* (microsporangiate strobili); MINOf: *M*. *chaohuensis* (megasporangiate strobili); WUXIm: *W*. *bistrobilata* (microsporangiate strobili); SUBLsm: *S*. *songziense* (microsporangiate strobili); SUBLsf: *S*. *songziense* (megasporangiate strobili); SUBLgf: *S*. *grabaui* (megasporangiate strobili); KOSV: *Kossoviella timanica*; YUGU: *Yuguangia ordinata*; CYMA: *Cymastrobus irvingii*; CLEV: *Clevelandodendron ohioensis*; LONG: *Longostachys latisporophyllus*; GUAN: *Guangdedendron micrum*; OMPR: *Omprelostrobus gigas*.

It has been stated that the body plan could control the size and organization of fertile structures in lycopsids^34^. To investigate the relationship between the dimensions of the strobili and the growth habit of the parent plant in Devonian lycopsids, representatives in Table 1 are plotted in Fig. 6. Both the maximum plant height and the size of strobili increased greatly from Middle to Late Devonian (Fig. 6, pins in different colors). Especially, a noteworthy increase occurred in the maximum widths of strobili, but not in the maximum lengths from Givetian to Famennian. *Omprelostrobus gigas* and *Guangdedendron micrum* (the two pins closest to the top-righthand corner in Fig. 6) possess larger strobili than other arborescent taxa. The stout strobili of *Clevelandodendron ohioensis* and *Cymastrobus irvingii* (the two in the top-lefthand corner in Fig. 6) are distinguished from other taxa, supporting the point “single-trunked isoëtaleans often produced large and wide strobili”^34^. Most of the others (Fig. 6, taxa in the lower-left part) possessed strobili of limited dimensions, despite their diversified plant size; therefore, the Devonian lycopsid strobili sizes could be relatively independent with their parent plants’ body plan.

The individual sizes of the tree lycopsids increased greatly during the Carboniferous period; similarly, the strobili of Carboniferous tree lycopsids, including some species of *Lepidostrobus* and *Flemingites*^19,35,36^, display larger sizes than most Devonian taxa^14,24,37^ and are usually considered as being pendulous during lifetime. Among the Devonian strobili listed here, *Changxingia longifolia* and *Guangdedendron micrum* are considered as pendulous^10,23^, while *Lilingostrobus chaloneri*, *Wuxia bistrobilata* and *Clevelandodendron ohioensis* were most likely erect^25,38,39^. *Omprelostrobus gigas* displays a curved fertile axis much slenderer than the strobilus attached to it, and thus the strobili of *O*. *gigas* are very likely to have been pendulous. It has been suggested that small-bodied herbaceous/pseudoherbaceous lycopsids usually had erect strobili and vice versa^25^. However, many Devonian arborescent lycopsids bore small strobili as previously discussed, and lack any direct evidence for a pendulous habit.

### Probable function of differentiated sporophyll laminae

**Fig. 7:**
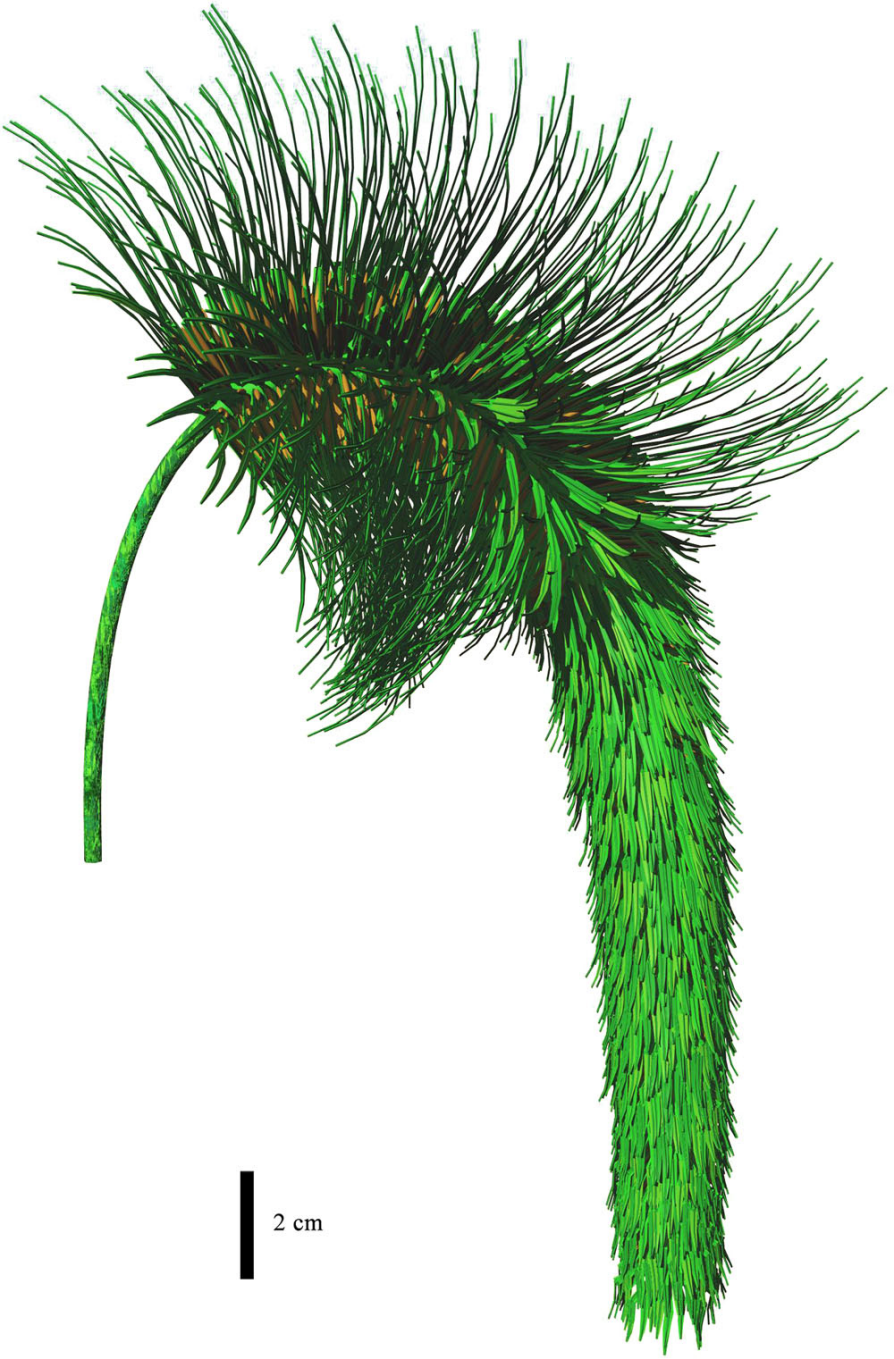
3-D reconstruction of a strobilus of *Omprelostrobus gigas* gen. et sp. nov., based on the type specimen shown in Fig. 2a.

A remarkable characteristic of *Omprelostrobus gigas* is the strobili divided into two portions according to the differentiated sporophyll laminae. Based on the type specimen, we tentatively reconstructed one strobilus as shown in Fig. 7. None of the preserved fertile axes of *O*. *gigas* show attachment of leaves, which is different from most other Late Devonian lycopsids including *Changxingia longifolia*, *Minostrobus chaohuensis* and *Guangdedendron micrum*, whose fertile axes possessed many microphylls^23,32,33^. The lack of vegetative leaves on fertile axes of *O*. *gigas* strobili may suggest additional photosynthetic activities in sporophylls for strobilus development, as the use of photosynthetic products in arborescent lycopsids is supposed to have been localized, and accordingly the sporangium nutrition benefited largely from photosynthesis of the sporophylls^18,40^.

Two Devonian strobili with prominent sporophylls, i.e., *Lilingostrobus chaloneri* and *Wuxia bistrobilata* (Table 1) could support the hypothesis of sporophyll photosynthesis. The obvious leaf vascular bundles of *L*. *chaloneri* are suggested as evidence for active photosynthesis in sporophylls^25^, while *W*. *bistrobilata* possessed similar sporophyll structure to *L*. *chaloneri* and may have had a comparable function. As for *O*. *gigas*, the strobili were probably pendulous, and the sporophylls on the proximal portion of the strobili have long laminae resembling those of *L*. *chaloneri* and *W*. *bistrobilata* that provided large photosynthetic areas. Moreover, the sporophylls in the distal portions also demonstrate a strong midvein similar to those of *L*. *chaloneri*, suggesting high photosynthetic capability.

We interpret the differentiation of *O*. *gigas* sporophyll laminae as a characteristic that facilitates photosynthesis: the radial orientation of the sporophylls on the proximal portion prevents self-shading, so that the sporophylls on the distal portion could be well exposed to sunlight (Fig. 7). Other possible explanations for such differentiation include different sporophyll shape as in bisporangiate strobili, successive developmental stages of sporophylls, benefitting the dispersal of spores or even offering defense against heavy rain, although support for these hypotheses is still required.

### Associated roots and affinity

Three major types of centralized root architecture have been reported in Devonian lycopsids, including the stigmarian type rhizomorph in the Famennian *Guangdedendron micrum*^10^, the cormose type rhizomorph in the Late Devonian *Leptophloeum rhombicum*^12^ and probably Givetian *Hoxtolgaya robusta*^41^, and the third type showing multiple isotomous root branches but lacking rootlets, in the Givetian *Longostachys latisporophyllus*^13^ and the Frasnian *Chamaedendron multisporangiatum*^42^.

The roots in Fig. 5 are frequently dichotomized and very similar to typical lycopsid roots^43^, while their close association with *Omprelostrobus gigas* plausibly hints at a former organic connection. The radial distribution of these roots suggests that they appear to be centralized roots extended from the base of the plant, rather than rootlets arranged in helices along rhizomorphic axes.

Some of the circular structures along the roots display similar appearance to the transverse sections of anatomically preserved, occasionally bifurcated rootlets^15,44^. These circular structures, however, differ from typical rootlet scars in the following ways. Firstly, they are easily removed by needles and thus may not be vascularized. Secondly, these structures are irregular in size and arrangement, and do not appear along the entire root branches but rather at the base. Furthermore, these dichotomized roots showed no evidence of rootlet adpression. Accordingly, these roots may represent a stage in the stigmarian-type rhizomorph evolution if the circular structures are related to rootlets; otherwise, they may represent a Famennian *Longostachys*-type root that lacked typical rootlets and passed through the F-F boundary.

Considering that there are only lycopsid organs in our fossil collection except for some branches of the seed fern *Cosmosperma polyloba*, we tend to think that all these lycopsids organs belong to *Omprelostrobus gigas*. The associated centralized roots, together with the large strobili and wide axes would strongly conform to the characteristics of the Order Isoёtales *sensu lato*^45^. However, we prefer not to assign *O*. *gigas* to any suborder or family due to the lack of clear in-situ spores.

### Ecological habit of some Late Devonian tree lycopsids

The axes of *Omprelostrobus gigas* and associated roots transversing the bedding plane suggest an autochthonous (in-situ) preservation. Recently reported localities containing in-situ Late Devonian tree lycopsid trunks include Svalbard in Norway and Xinhang in China^9,10^. In addition to providing evidence for early forest ecosystems, these upright trunks could represent a flooded environment: such a special taphonomic phenomenon requires a high rate of deposition, caused by currents carrying lots of sediments^46^. Sporadically flood events together with burial of sediments may lead to severe disturbance and exclusion of other plants^18^. However, some characteristics may indicate the adaptability of Devonian tree lycopsids to such an environment.

*Omprelostrobus gigas* and *Guangdedendron micrum* possess the first and the second largest strobili among Devonian arborescent lycopsids (Fig. 6 and Table 1). These two plants were small trees but had strobili of similar dimensions to some Carboniferous taxa, e.g., some species of *Lepidostrobus*^19^, whose parent plants were probably gigantic. The relatively large strobili of *O*. *gigas* and *G*. *micrum* may reflect increased reproductive investment in propagule production. In addition, *G*. *micrum* is thought to have been monocarpic^10^; such a trait, together with the large commitment to reproduction, matches the plant strategy of ruderal-type^47,48^ and favors persistence under conditions of frequent disturbance. On the other hand, at the Fanwan Section the pteridosperm *Cosmosperma polyloba* shows stems obliquely crossing the bedding plane and well-preserved delicate leaves^27^ as probable evidence of in-situ preservation together with *O*. *gigas*. Many of the early seed plants were considered to be “opportunists” by previous researchers^49–51^. The similarity with *G*. *micrum* and co-occurrence with *C*. *polyloba* support the opinion that *O*. *gigas* could adapt to the disturbed niches. The adaptation to disturbance would allow some Devonian tree lycopsids, including *O*. *gigas* and *G*. *micrum*, to flourish in the coastal lowland environment with sporadically flood events.

The Wutong Formation on the South China Plate containing *Omprelostrobus gigas* and *Guangdedendron micrum* represents the coastal environment in a tropical area^10^. The roots of *G*. *micrum* and those associated with *O*. *gigas* are much smaller when compared to the shoots, suggesting low root: shoot ratio suitable for environments with high temperature and sufficient water supply^52^. The Xinhang forest was mainly composed of *G*. *micrum*^10^, while the Frasnian Svalbard forest in the palaeoequatorial wetland zone also consisted of lycopsids^9^. We consider that the tropical coastal forests in the Late Devonian were probably dominated by tree lycopsids.

## Methods

The fossil plant is mostly preserved as brownish adpressions in yellow argillaceous siltstone containing tiny crystals of quartz and micas, and sometimes associated with branches of the seed plant *Cosmosperma polyloba*. Axes casts and roots penetrating across the bedding plane indicate an autochthonous (in-situ) preservation. Steel needles were used for dégagement, and a digital camera and a stereoscope were employed for photographs. Measurements are based on both fossil specimens and photographs. Photos were processed with Photoshop CC software. Data plot was generated by PAST3 software. CorelDRAW X7 and Plantfactory 2014 were used for line drawing and plant reconstruction, respectively. All the specimens are housed at the Department of Geology, Peking University, Beijing, China.

### Reporting summary

Further information on research design is available in the Nature Research Reporting Summary linked to this article.

## Supplementary information

Supplementary Information

Reporting Summary

## Data Availability

All data generated or analyzed during this study are included in this published article (and its supplementary information files). Fossil specimens used in this study (PKUB19301-PKUB19309, PKUB19310A, B, PKUB19311) are excavated from the Upper Devonian (Famennian) at Fanwan Village, Hongqiao Town, Changxing County, Zhejiang Province, China, and are housed at the Department of Geology, Peking University, Beijing, China.
