# Supplementary material for: A Late Devonian tree lycopsid with large strobili and isotomous roots
Source: Commun Biol. 2022 Sep 15;5:966. doi: 10.1038/s42003-022-03934-4 (PMC9478126; doi:10.1038/s42003-022-03934-4)
Supplement: Supplementary file 1 — Supplementary Information [file 42003_2022_3934_MOESM1_ESM.pdf]

A Late Devonian tree lycopsid with large strobili and isotomous roots

Le Liu[1], De-Ming Wang[2]*, Yi Zhou[2], Min Qin[3], David K Ferguson[4] and Mei-Cen Meng[5]

[1] College of Geoscience and Surveying Engineering, China University of Mining and Technology (Beijing), Beijing 100083, China.

[2] Key Laboratory of Orogenic Belts and Crustal Evolution, School of Earth and Space Sciences, Peking University, Beijing 100871, China.

[3] Institute of Geology and Paleontology, Linyi University, Linyi 276000, China

[4] Department of Palaeontology, University of Vienna, Vienna 1090, Austria

[5]Science Press, China Science Publishing & Media Ltd., Beijing 100717, China.

*Author for correspondence: dmwang@pku.edu.cn, +86 10 6276-1095

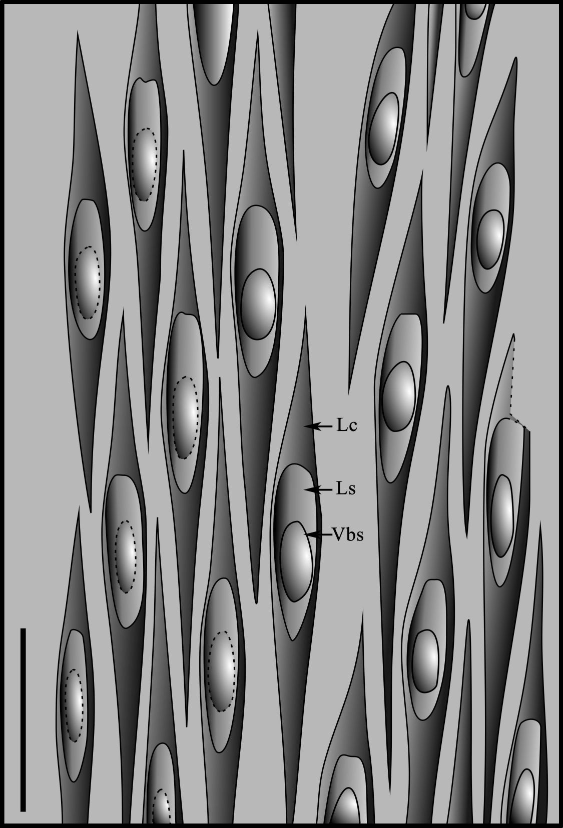
Lc
Ls
Vbs

Supplementary Figure 1. Interpretative line drawing of the leaf cushions displayed in Fig. 1h. Lc: leaf cushion. Ls: leaf scar. Vbs: vascular bundle scar. Dashed lines indicating speculated position of vascular bundle scar. Scale bar: 5 mm.
