# Supplementary material for: A Late Devonian tree lycopsid with large strobili and isotomous roots
Source: Commun Biol. 2022 Sep 15;5:966. doi: 10.1038/s42003-022-03934-4 (PMC9478126; doi:10.1038/s42003-022-03934-4)
Supplement: Supplementary file 2 — Reporting Summary [file 42003_2022_3934_MOESM2_ESM.pdf]

nature research

Corresponding author(s): Deming Wang

Last updated by author(s): Aug 16, 2022

Reporting Summary

Nature Research wishes to improve the reproducibility of the work that we publish. This form provides structure for consistency and transparency in reporting. For further information on Nature Research policies, see our Editorial Policies and the Editorial Policy Checklist.

Statistics

For all statistical analyses, confirm that the following items are present in the figure legend, table legend, main text, or Methods section.

n/a	Confirmed
☒	☐ The exact sample size (n) for each experimental group/condition, given as a discrete number and unit of measurement
☒	☐ A statement on whether measurements were taken from distinct samples or whether the same sample was measured repeatedly
☒	☐ The statistical test(s) used AND whether they are one- or two-sided<br>*Only common tests should be described solely by name; describe more complex techniques in the Methods section.*
☒	☐ A description of all covariates tested
☒	☐ A description of any assumptions or corrections, such as tests of normality and adjustment for multiple comparisons
☒	☐ A full description of the statistical parameters including central tendency (e.g. means) or other basic estimates (e.g. regression coefficient) AND variation (e.g. standard deviation) or associated estimates of uncertainty (e.g. confidence intervals)
☒	☐ For null hypothesis testing, the test statistic (e.g. F, t, r) with confidence intervals, effect sizes, degrees of freedom and P value noted<br>*Give P values as exact values whenever suitable.*
☒	☐ For Bayesian analysis, information on the choice of priors and Markov chain Monte Carlo settings
☒	☐ For hierarchical and complex designs, identification of the appropriate level for tests and full reporting of outcomes
☒	☐ Estimates of effect sizes (e.g. Cohen's d, Pearson's r), indicating how they were calculated

Our web collection on statistics for biologists contains articles on many of the points above.

Software and code

Policy information about availability of computer code

Data collection: Not applicable

Data analysis: Not applicable

For manuscripts utilizing custom algorithms or software that are central to the research but not yet described in published literature, software must be made available to editors and reviewers. We strongly encourage code deposition in a community repository (e.g. GitHub). See the Nature Research guidelines for submitting code & software for further information.

Data

Policy information about availability of data

All manuscripts must include a data availability statement. This statement should provide the following information, where applicable:

- Accession codes, unique identifiers, or web links for publicly available datasets
- A list of figures that have associated raw data
- A description of any restrictions on data availability

All the used data for this study are embedded in the manuscript and supplementary files.

Field-specific reporting

Please select the one below that is the best fit for your research. If you are not sure, read the appropriate sections before making your selection.

☐ Life sciences ☐ Behavioural & social sciences ☒ Ecological, evolutionary & environmental sciences

For a reference copy of the document with all sections, see nature.com/documents/nr-reporting-summary-flat.pdf

Ecological, evolutionary & environmental sciences study design

All studies must disclose on these points even when the disclosure is negative.

Study description	A new Devonian lycopsid is described based on new fossil collections, with its characteristics and habits compared to related taxa and discussed.
Research sample	The research sample is a fossil collection of about 30 specimens containing the Devonian lycopsid with identifiable characteristics.
Sampling strategy	All the available fossil specimens were used for the study.
Data collection	Le Liu performed the experiment and collected the data. The dimensions of fossil plant were measured during observation.
Timing and spatial scale	The data were collected during the experiment in Jul. to Nov., 2020.
Data exclusions	No data were excluded from the analyses.
Reproducibility	The characteristics of the fossil plant are supported by multiple specimens.
Randomization	Not applicable
Blinding	Not applicable

Did the study involve field work? ☒ Yes ☐ No

Field work, collection and transport

Field conditions	Field work was only used to collect fossil specimens and thus not affected by field conditions.
Location	The studied fossils were colleceted in 13th bed of Fanwan Section at Changxing County, Zhejiang Province, China (30°58′4″N and 120°02′34″E).
Access & import/export	Not applicable
Disturbance	No disturbance is caused by the study.

Reporting for specific materials, systems and methods

We require information from authors about some types of materials, experimental systems and methods used in many studies. Here, indicate whether each material, system or method listed is relevant to your study. If you are not sure if a list item applies to your research, read the appropriate section before selecting a response.

Materials & experimental systems

n/a	Involved in the study	
☒	☐	Antibodies
☒	☐	Eukaryotic cell lines
☐	☒	Palaeontology and archaeology
☒	☐	Animals and other organisms
☒	☐	Human research participants
☒	☐	Clinical data
☒	☐	Dual use research of concern

Methods

n/a	Involved in the study	
☒	☐	ChIP-seq
☒	☐	Flow cytometry
☒	☐	MRI-based neuroimaging

Palaeontology and Archaeology

Specimen provenance	The fossil specimens were collected by authors and their collaborators and no permit is needed for the field work.

Specimen deposition	All the specimens are deposited in the Department of Geology, Peking University.
Dating methods	No new dates are provided.

☒ Tick this box to confirm that the raw and calibrated dates are available in the paper or in Supplementary Information.

Ethics oversight	No ethical approval was required for this study.

Note that full information on the approval of the study protocol must also be provided in the manuscript.
